# Piwi in the stem cell niche regulates nurse cell number and oocyte specification

**DOI:** 10.17912/micropub.biology.000327

**Published:** 2020-11-18

**Authors:** Gina Zhu, Lauren E Gonzalez, Haifan Lin

**Affiliations:** 1 Yale Stem Cell Center, Yale School of Medicine, New Haven, CT 06519, USA; 2 Yale College, New Haven, CT 06511, USA; 3 Department of Genetics, Yale School of Medicine, New Haven, CT 06519, USA; 4 Department of Cell Biology, Yale School of Medicine, New Haven, CT 06519, USA

**Figure 1. Knockdown of piwi expression in terminal filament and cap cells, but not posterior escort cells, results in GSC proliferation and differentiation defects:**
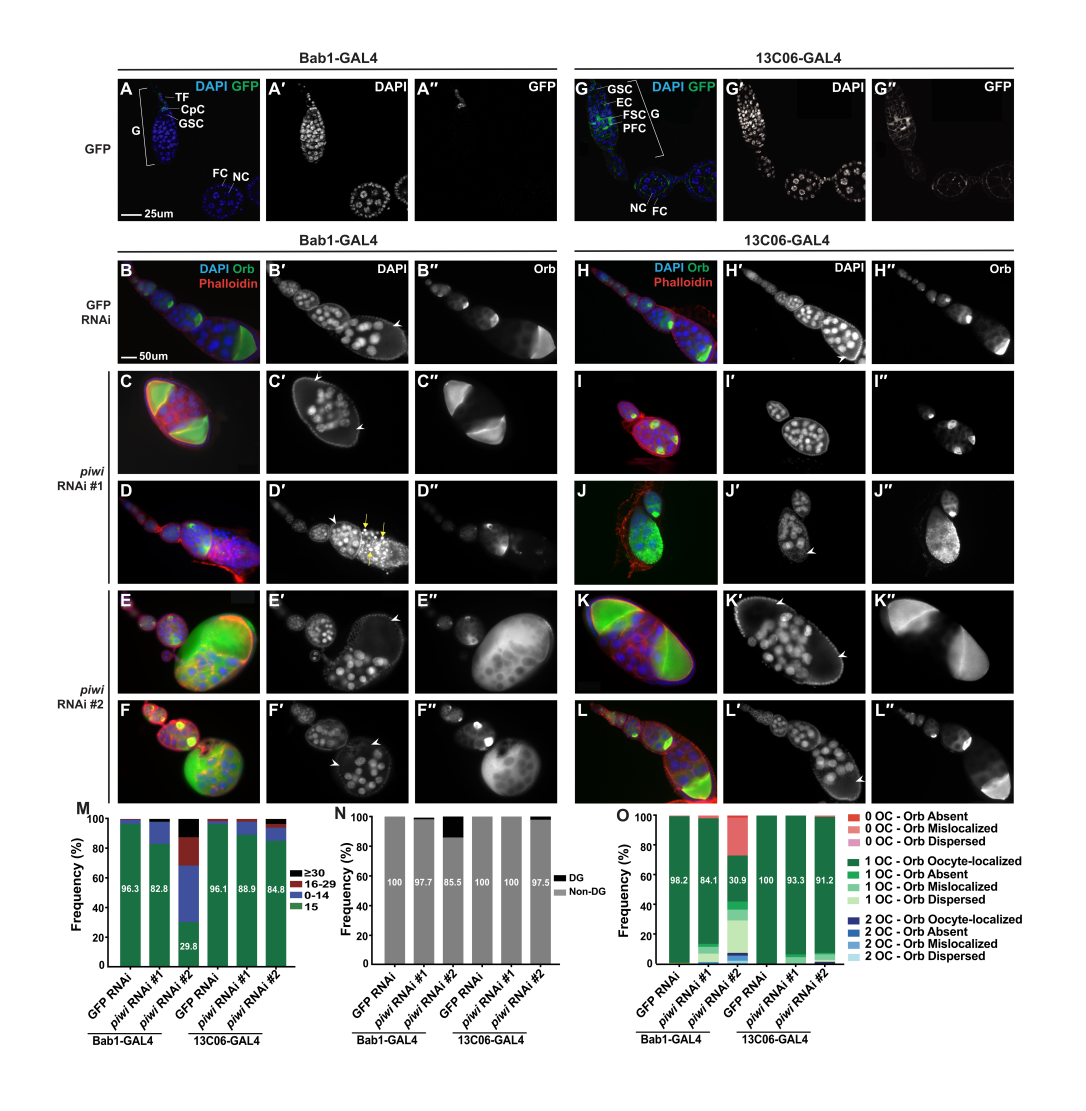
**(A – A′′)** *Bab1-GAL4* driving *UASp-GFP* demonstrates that the driver is expressed in terminal filament and cap cells. **(B – F′′)** *Bab1-GAL4 RNAi* experiments show that *piwi* depletion in terminal filament and cap cells causes dramatic morphological defects. DAPI marks nuclei, Orb is an oocyte-specific protein, and Phalloidin marks F-actin structures, including ring canals. Presumptive oocytes, identified by DAPI and Phalloidin staining, are indicated with white arrow heads. Degenerate nurse cells, identified by pyknotic nuclei, are indicated with yellow arrows. **(G – G′′)** *13C06-GAL4* driving *UASp-GFP* demonstrates that the driver is expressed in posterior escort cells, follicle stem cells, and prefollicle cells. **(H – L′′)** *13C06-GAL4* RNAi experiments show that *piwi* depletion in posterior escort cells, follicle stem cells, and prefollicle cells causes some morphological defects. DAPI staining marks nuclei, Orb is an oocyte-specific protein, and Phalloidin marks F-actin structures, including ring canals. Presumptive oocytes, identified by DAPI and Phalloidin staining, are indicated with white arrow heads. (**M)** The frequency of egg chambers with 15, 0-14, 16-29, and a tumorous (≥ 30) number of non-degenerate nurse cells per egg chamber for each genotype. *Piwi-bab1*KD#2 resulted in a decrease in the number of egg chambers with the expected 15 nurse cells. *Piwi-*13C06KDresulted in relatively few egg chambers with defects in nurse cell number. **(N)** The frequency of egg chambers that had non-degenerate and degenerate nurse cells. Both *piwi-bab1*KDand *piwi-*13C06KDresulted in relatively few egg chambers with degenerate nurse cells. **(O)** The frequency of egg chambers with zero, one, two, or three presumptive oocytes (OC) and with oocyte-localized, absent, mislocalized, or dispersed Orb staining. *Piwi-bab1*KD#2 resulted in a decrease in the number of egg chambers with only one presumptive oocyte and oocyte-localized Orb staining, while *piwi-*13C06KDresulted in relatively few egg chambers with defects in oocyte specification or Orb localization. The number of egg chambers counted for each genotype is as follows: *bab1- GAL4>GFP* RNAi = 109; *bab1-GAL4>piwi* RNAi #1 = 88; *bab1-GAL4>piwi* RNAi #2 = 94; *13C06-GAL4>GFP* RNAi = 1210; *13C06-GAL4>piwi* RNAi #1 = 90; *13C06-GAL4>piwi* RNAi #2 = 79. CpC = cap cell, DG = degenerate, EC = escort cell, FC = follicle cell, FSC = follicle stem cell, G = germarium, GSC = germline stem cell, NC = nurse cell, OC = oocyte, PFC = prefollicle cell, TF = terminal filament.

## Description

In the *Drosophila* ovary*,* the proliferation of germline cells and differentiation of the oocyte is informed by cues from a variety of ovarian somatic cell (OSC) types. In particular, the stem cell niche, composed of cap cells, terminal filament cells, and escort cells, directly signals to germline stem cells (GSCs) to promote their self-renewal and differentiation into cystoblasts. Cap cells express Decapentaplegic (Dpp), which signals to the germline to promote proliferation and maintenance of GSCs (Xie and Spradling 1998, Chen and McKearin 2003, Song *et al.* 2004). Subsequently, the differentiation of cystoblasts and their four synchronous divisions with incomplete cytokinesis to form 16-cell cysts depends on the repression of Dpp by Wnt, EGF, and Hedgehog signaling from escort cells (Liu *et al.* 2010, Luo *et al.* 2015, Mottier-Pavie *et al.* 2016, Huang *et al.* 2017). The germline cyst then becomes encased in somatic follicle cells to form an egg chamber, in which one germline cell is the oocyte and the other 15 are polyploid nurse cells. Follicle cells impact multiple aspects of germline differentiation throughout the rest of oogenesis, including polarization of the oocyte guided by JAK/STAT and Notch signaling (reviewed in Riechmann and Ephrussi 2001).

Early studies aimed at identifying genes involved in the balance of GSC maintenance and differentiation uncovered *piwi* (Cox *et al.* 1998, Cox *et al.* 2000). Clonal analysis showed that *piwi* expression in somatic, but not germline, cells is required for GSC maintenance (Cox *et al.* 1998). This led to the suggestion that Piwi mainly functions on GSCs through the stem cell niche. Mechanistic studies into Piwi and its associated small noncoding Piwi-interacting RNAs (piRNA), which guide Piwi to target sequences, have since focused on their role in transposon suppression. There is also a growing body of evidence that Piwi regulates some non-transposon gene expression programs during development (reviewed in Ozata *et al.* 2019).

Tissue-specific knockdown using the *UAS/GAL4* system now provides an opportunity to dissect the function of Piwi in specific cell types of the ovary. Early studies that used this approach focused on the proliferation and differentiation of germline cells within the germarium, and revealed that depletion of *piwi* in escort cells results in an over-proliferation of undifferentiated GSCs into “GSC tumors” (Jin *et al.* 2013, Ma *et al.* 2014). In our study focusing on mid-stage egg chambers, after depleting *piwi* in all OSCs using *traffic jam-GAL4* (“*piwi*-sKD”), we observed defects in nurse cell number, oocyte number, and oocyte specification (Gonzalez *et al.* 2020). Because the aberrant egg chambers often contained improper numbers of nurse cells (but rarely in multiples of 15) and could contain from 0 to 3 “oocytes” (determined by morphology and localization of the oocyte marker protein Orb), we concluded that this phenotype was more likely due to improper germline proliferation and differentiation than improper encapsulation of the germline by follicle cells. Here, we further investigate whether these defects are due to loss of Piwi activity in terminal filament and cap cells (using *bab1-GAL4,* Figure 1A-A′′), and/or in escort cells (using *13C06-GAL4*, Figure 1G-G′′).

Depletion of *piwi* in terminal filament and cap cells via *bab1-GAL4* (herein referred to as *piwi*–*bab1*KD, Figure 1B-1F′′) resulted in egg chambers with defects in nurse cell number and oocyte specification, partially phenocopying *piwi*-sKD driven by *tj-GAL4*, which depletes *piwi* in all OSCs*.* 96.3% of the *GFP-bab1*KD egg chambers contained the expected 15 nurse cells, while only 82.8% and 29.8% of egg chambers in *piwi*–*bab1*KD #1 and *piwi-bab1*KD #2, respectively, contained exactly 15 nurse cells (Figure 1M). However, unlike depletion of *piwi* in all somatic cells, very few egg chambers contained either tumorous (Figure 1M) or degenerate (Figure 1N) germline cells. Thus, *piwi* depletion in terminal filament and cap cells explains some, but not all, of the nurse cell number defects caused by *piwi*-sKD.

We also observed defects in oocyte specification upon *piwi-bab1*KD (Figure 1B-1F′′). Only 84.1% and 30.9% of *piwi*– *bab1*KD #1 and *piwi*–*bab1*KD #2 egg chambers, respectively, contained egg chambers with a single oocyte at the posterior of the egg chamber and displaying Orb accumulation (Figure 1O). Within egg chambers that contained one presumptive oocyte (96.6% and 65.8% of egg chambers in *piwi*–*bab1*KD #1 and *piwi-bab1*KD #2, respectively), the most common abnormality in oocyte phenotype was a dispersal of Orb throughout the egg chamber (Figure 1O), indicating that oocyte specification is impaired upon *piwi*–*bab1*KD.

Notably, *piwi-bab1*KD #1 had a milder effect on both nurse cell number and oocyte specification than *piwi*–*bab1*KD #2. We had previously shown that *piwi-*RNAi #2 more strongly depletes *piwi* mRNA levels compared to *piwi-*RNAi #1 when driven in all somatic cells (Gonzalez *et al.* 2020), so this may reflect that a threshold level of *piwi* is necessary in the stem cell niche for this function.

We then investigated the effect of depleting *piwi* in posterior escort cells, follicle stem cells, and pre-follicle cells using *13C06-GAL4* (Figure 1G-1L′′). Previous studies had shown a major role for Piwi in escort cells in regulating cystocyte differentiation (Jin *et al.* 2013, Ma *et al.* 2014), so we were surprised to observe relatively few egg chambers with defects in nurse cell number and/or oocyte specification following *piwi*-13C06KD (Figure 1M-1O). Our result indicates that Piwi activity in posterior escort cells and follicle stem cells may not regulate nurse cell number and oocyte specification, and suggests that the GSC tumors described by previous studies may not be linked to the mid-stage germline defects we have described. However, we depleted *piwi* using *13C06-GAL4*, which is expressed weakly in anterior escort cells but strongly in posterior escort cells (Sahai-Hernandez and Nystul 2013), while previous studies used *c587-GAL4*, which expresses more strongly in anterior escort cells (Song *et al.* 2004), so it is possible that Piwi’s escort cell function is primarily in anterior escort cells.

Altogether, these results indicate that Piwi functions within the stem cell niche, and perhaps mostly within terminal filament and cap cells, to regulate germline proliferation and differentiation within the germarium, and that this has long-lasting developmental effects on egg chambers throughout oogenesis. The proliferation of germline cells and specification of the oocyte are temporally and spatially separate from the GSC-niche interaction, so the observation that gene expression in terminal filament and cap cells can influence these processes is striking. It suggests that the niche not only regulates the self-renewal of GSCs and their immediate differentiation into cystoblasts, but also modulates their developmental potential for processes that occur later in oogenesis. Alternatively, because *bab1-GAL4* is also active in somatic cells of the larval gonad (Cho *et al.* 2018), our results may reflect a requirement for somatic *piwi* during gonadogenesis for the subsequent organization, proliferation, and specification of germline cells in the adult ovary.

Piwi regulates gene expression at both the transcriptional level (Brower-Toland *et al.* 2007, Wang & Elgin 2011, Sienski *et al.* 2012) and the post-transcriptional level (Robine *et al.* 2009, Saito *et al.* 2009, Klein *et al.* 2016), so further investigations should seek to understand which gene expression programs Piwi directly regulates to influence these oogenic processes. The observation that *piwi* depletion in somatic cells impacts the proliferation and specification of germline cells suggests that Piwi regulates the expression of genes involved in soma-to-germline signaling. Piwi has previously been shown to mediate Dpp signaling to regulate GSC maintenance (Jin *et al.* 2013, Ma *et al.* 2014), and the piRNA biogenesis factor Yb has been suggested to interact with the Notch pathway to regulate very similar nurse cell and oocyte phenotypes to those we have described upon *piwi*-sKD (Johnson *et al.* 1995). Future studies should investigate whether Piwi in the stem cell niche regulates the expression of genes in the Dpp, Notch, or other signaling pathways. These new findings would add to the growing evidence that the biological function of the PIWI/piRNA pathway extends well beyond the suppression of transposons.

## Methods

**Drosophila husbandry and genetics**

All *Drosophila* stocks were raised on standard agar/molasses medium and raised at 25°C for experiments. *Bab1-GAL4* (BDSC stock #6802) was used to express UASpconstructs in all cap cells and terminal filament cells throughout development, and *13C06-GAL4* (BDSC stock #47860) was used to express UASp constructs in posterior escort cells, follicle stem cells, and prefollicle cells. *UASp-GFP* (BDSC #1521) was used to verify the expression pattern of *GAL4* lines. Two anti-Piwi RNAi lines (“*piwi* RNAi #1” and “*piwi-*RNAi #2” in this study) were used to knock down *piwi* expression. *Piwi-siRNA #1* targets exon 2 of the *piwi* mRNA and is BDSC stock #37483; *piwi siRNA #2* targets exon 3 of the *piwi* mRNA and was a gift from T. Xie, Stowers Institute For Medical Research, Kansas City, MO. *GFP*-RNAi (BDSC stock #41550) was used as a negative control. To generate *GAL4/UASp* flies for analysis, two males carrying the *GAL4* driver were crossed with three virgin females carrying the *UASp* construct. *GAL4/UASp* females were aged for two to three days in a ratio of 2:1 with *w1118* males prior to ovary dissection.

**Immunostaining**

Ovaries from 2-3 day old females were dissected in 1X PBS and fixed with 200 μL of the following fixing solution (v/v%): PBS (89.5%), 10% Nonidet P-40 (5%), 37% formaldehyde (5.5%). The fixed ovaries were then washed 3 times for 15 minutes each in PBST (PBS and 0.2% Triton X-100). Ovaries were then blocked overnight in 5% NGS at 4^o^C, followed by incubation overnight at 4^o^C in primary antibody diluted in PBST + 2% NGS. Samples were washed three times in PBST and incubated in secondary antibodies overnight at 4^o^C. Samples were washed three times with PBST, stained with DAPI (1:500) and Phalloidin (1:200, ThermoFisher, #R415) for 15 minutes and mounted in Vectashield mounting media (Vector Labs, #H1000).

We used mouse anti-Orb 4H8 (1:300, DSHB) to visualize Orb localization. The following conjugated secondary antibodies were used, all at 1:500 dilutions: The Alexa 488-conjugated goat anti-mouse antibody, the Alexa 555- conjugated goat anti-mouse. Orb, Phalloidin, and DAPI were used qualitatively and quantitatively characterize the observed phenotypes of the egg chambers from all crosses.

**Microscopy and phenotypic characterization**

To observe the GAL4 expression pattern, confocal images of DAPI stained *bab1-GAL4>UASp-GFP* and *13C06-GAL4>UASp-GFP* ovaries were taken using Leica TCS SP5 Confocal Laser Scanning Microscope. *Piwi*-knockdown samples were analyzed on the ZEISS Axio Imager2 for quantitative and qualitative characterizations.

DAPI and Phalloidin staining was used to identify nurse cells and oocytes. To more accurately quantify the effect of *piwi*– *bab1*KD and *piwi*-13C06KD on the number of nurse cells, we created four categories to describe how many nurse cells were in an egg chamber: 15 normal nurse cells; 0-14 normal nurse cells; 16-29 normal nurse cells; and ≥30 normal nurse cells, which we referred to as a tumorous number. Degenerate nurse cells were identified by the presence of bright pyknotic nuclei. Any large area of cytoplasm without a polyploid nucleus was considered a presumptive oocyte. We quantified how many egg chambers had zero, one, two, or three presumptive oocytes. We also characterized the localization of the Orb staining based on its relative position with the presumptive oocyte. We created four categories of Orb’s localization pattern: absent, oocyte-localized, mislocalized, and dispersed. If the egg chamber had no trace of Orb staining, the pattern was considered absent. If the Orb staining localized with the presumptive oocyte(s) it was designated oocyte-localized; but if the Orb staining was not localized to the presumptive oocyte and formed discrete patches, it was considered mislocalized. If the Orb staining filled the entire egg chamber, its pattern was considered dispersed.
